# Cryptococcal pericarditis in a heart transplant recipient

**DOI:** 10.1111/tid.13366

**Published:** 2020-06-26

**Authors:** Sarah Mann, Farrell Tobolowsky, Suneet Purohit, Andres Henao-Martínez, Valida Bajrovic, Poornima Ramanan, Eugene Wolfel, Prateeti Khazanie, Michelle Barron, Nancy Madinger, Esther Benamu

**Affiliations:** 1 Division of Infectious Diseases, University of Colorado School of Medicine, Aurora, Colorado, USA; 2 Division of Cardiology, University of Colorado School of Medicine, Aurora, Colorado, USA

**Keywords:** *Cryptococcus neoformans*, heart transplant, pericarditis

## Abstract

We present a case of *Cryptococcus neoformans* pericarditis in a cardiac transplant recipient. This article reviews the diagnosis, treatment, and complications of cryptococcosis specifically in transplant patients. While pericarditis is a rare manifestation of *Cryptococcus* infection, this case highlights that cryptococcosis should be considered in the differential diagnosis for solid organ transplant and immunocompromised patients presenting with pericardial effusions.

## 1 | INTRODUCTION

A 52-year-old man with a past medical history of orthotopic heart transplantation 1 year prior to presentation due to a genetic cardiomyopathy on chronic immunosuppression with prednisone, tacrolimus, and mycophenolate presented to the hospital with worsening fatigue and syncope. The patient had a history of early post-transplant rejection (within a month of transplant) treated with antithymocyte globulin. Two weeks prior to hospital admission, an endomyocardial biopsy showed no evidence of cellular or antibody-mediated rejection. However, the patient was diagnosed with amiodarone thyroiditis and was started on methimazole as well as high-dose prednisone.

The patient endorsed a 3-month history of fatigue, but denied fevers, chills, chest pain, palpitations, cough, headaches, visual problems, photophobia, neck stiffness, or skin lesions. Born in San Francisco, he previously lived in Germany until moving to Colorado. He worked as a construction engineer and had a parakeet from ages 11 to 16 years old.

**FIGURE 1:**
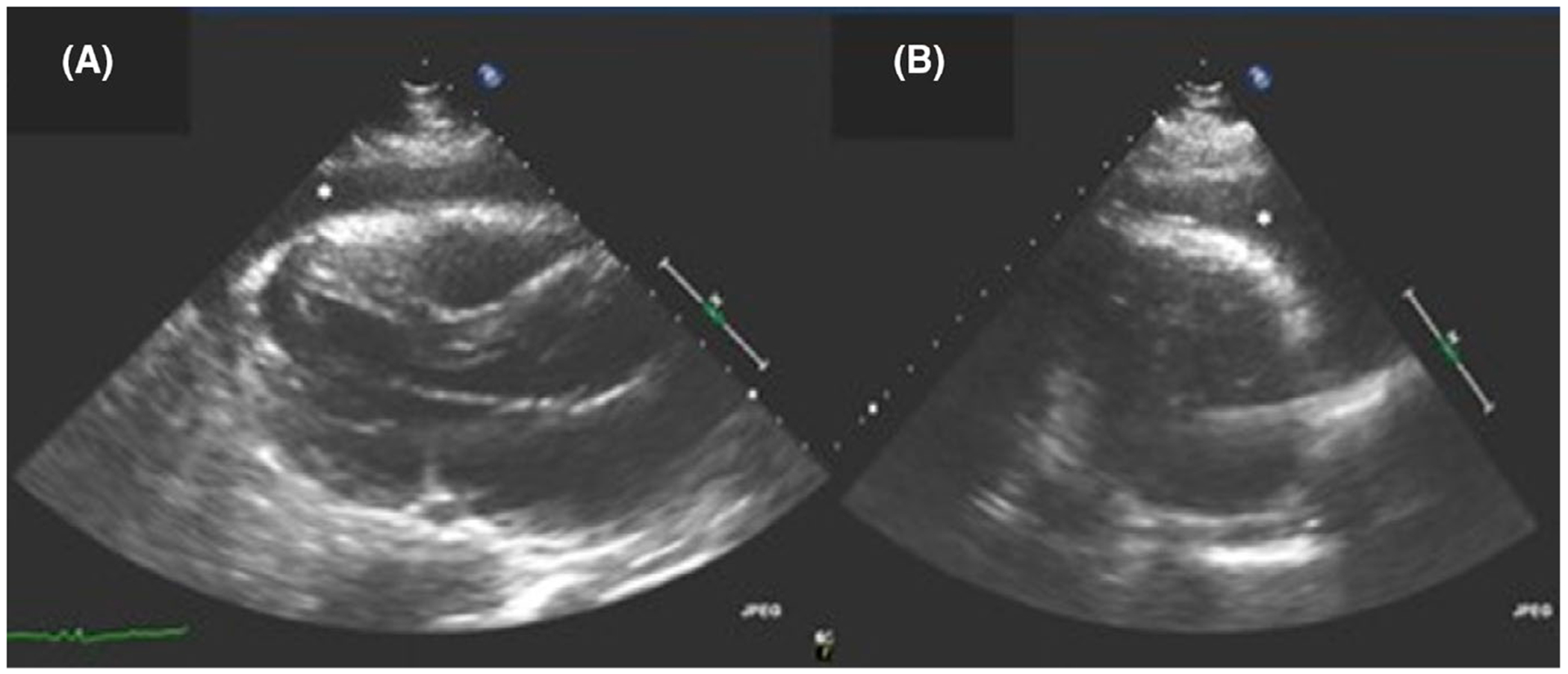
A, An anterior pericardial effusion marked by the * that was seen on the parasternal long axis views on transthoracic echocardiography. B, An anterior pericardial effusion marked by the * that was seen on the parasternal short axis views on transthoracic echocardiography

**FIGURE 2:**
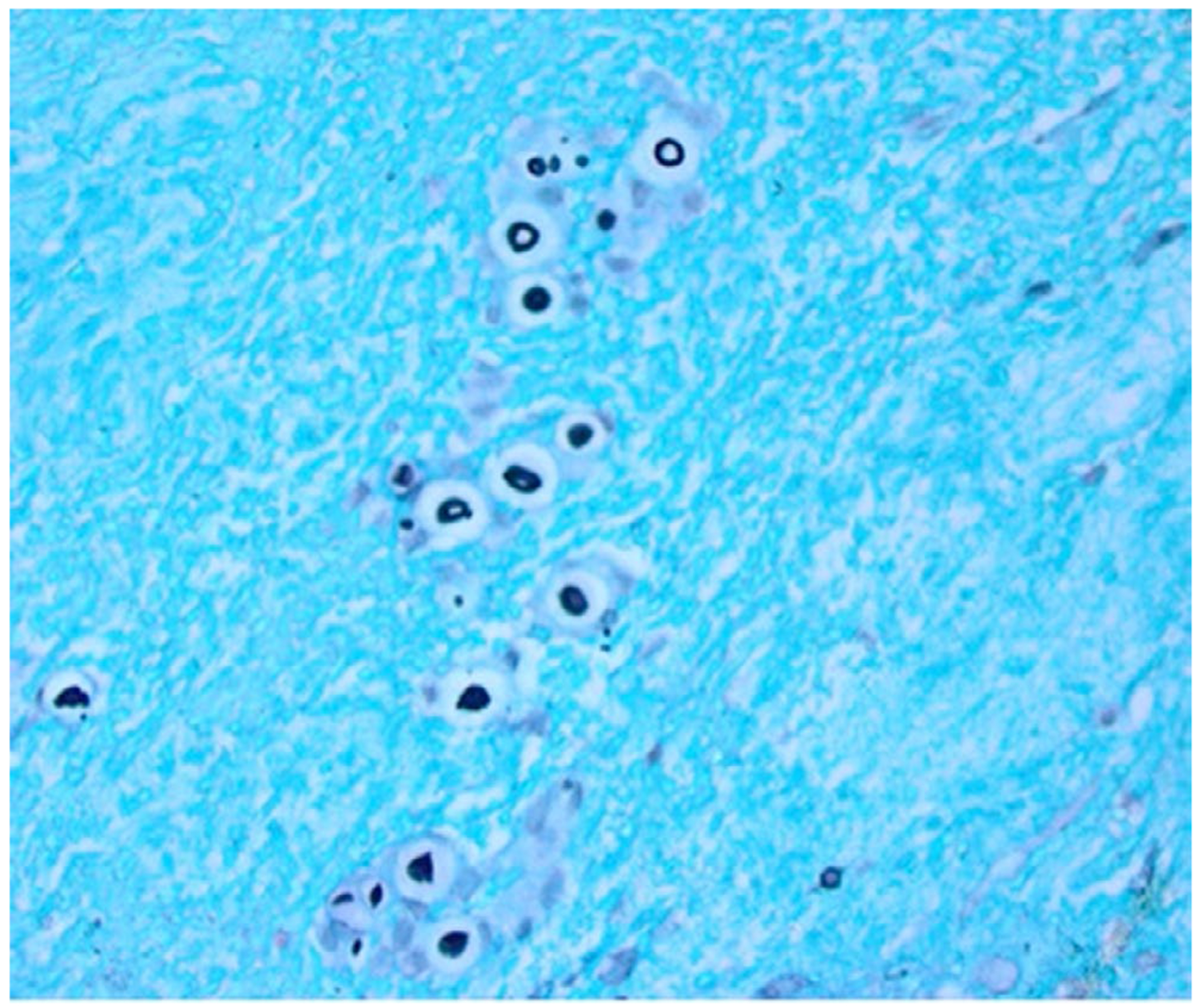
Pericardial biopsy with GMS stain revealing fungal organisms identified as Cryptococcus

His physical examination on admission was notable for elevated jugular venous pressure and wheezing in the right lung base. Electrocardiogram demonstrated diffuse low QRS voltages, sinus tachycardia with premature atrial complexes, and left posterior fascicular block. Echocardiogram revealed normal left and right ventricular systolic function and a rapidly enlarging pericardial effusion, which was not present on his most recent study 4 days prior (Figure 1). A pericardiocentesis was performed, and after 48 hours of incubation, cultures were positive for *Cryptococcus neoformans*. Subsequent echocardiograms showed a persistent and loculated pericardial effusion. Cardiothoracic surgery was consulted and drained the fluid through a pericardial window. Histopathology of the pericardial tissue revealed numerous yeasts morphologically compatible with *Cryptococcus* sp. (Figure 2). While *Cryptococcus* sp. was predominantly found within the proteinaceous lining of the pericardial tissue, some organisms appeared to be present within the pericardial tissue as well. Fungal culture from the pericardium also grew *C neoformans*.

Blood cultures, Human Immunodeficiency Virus (HIV) serology, cerebrospinal fluid fungal culture, and cryptococcal antigen in serum and spinal fluid were negative. Lumbar puncture demonstrated an opening pressure of 22 cm H_2_O with 0 white blood cells and normal protein. Computed tomography of the chest, abdomen, and pelvis and brain magnetic resonance imaging were unremarkable.

Treatment with liposomal amphotericin B (L-Amb) 3 mg/kg/d and flucytosine 100 mg/kg was initiated; doses of mycophenolate and tacrolimus were decreased and steroid doses were progressively tapered. Due to volume overload and progressive renal dysfunction, he was transitioned to high-dose fluconazole monotherapy (equivalent of 800 mg daily renally adjusted) after 10 doses of L-Amb over 3 weeks. Right and left heart catheterization demonstrated elevated right and left sided filling pressures with diastolic equalization, respirophasic concordance, and low cardiac output consistent with cardiogenic shock due to restrictive cardiomyopathy. Repeat echocardiograms demonstrated no pericardial effusion.

The patient eventually became anuric requiring hemodialysis. A cardiac MRI was performed, noting a thick pericardium with pericardial adhesions to the right ventricular free wall. However, there was no interventricular dependence or other hemodynamic changes to suggest constrictive pericarditis. Redo heart transplantation was considered but deemed too high risk due to acute infection.

He was discharged on fluconazole 400 mg daily (renally adjusted) for maintenance therapy and at the time of publication of this manuscript, is continuing with close monitoring. Serial echocardiograms have continued to demonstrate resolution of the pericardial effusion.

## 2 | DISCUSSION

*Cryptococcus neoformans*, an encapsulated basidiomycetous yeast, commonly causes disease in immunocompromised patients and is the third leading fungal infection in solid organ transplant (SOT) recipients.^1^ While any organ system can become infected, central nervous system and pulmonary infections are the most common.^1–3^ Pericardial cryptococcal infection, however, is rare, and few case reports have been described in the literature (see Tables 1 and 2).^4–13^ Cryptococcal pericarditis has been described in patients with SOT as well as non-transplant patients (see Tables 1 and 2).^4–13^ Among non-transplant cases, most patients with cryptococcal pericarditis had an underlying comorbidity such as HIV, metastatic prostate cancer, Hodgkin’s disease, or intravenous drug use that likely predisposed them to the disease (see Table 2). These cases did not describe locus minoris resistentiae, an injured body region that is vulnerable to disease, leading to the development of cryptococcal pericarditis. Most patients clinically improved, but cryptococcal pericarditis can be fatal.

With the advent of antiretroviral medications, cryptococcal infection is now more common in transplant patients than the traditional HIV cohort in the United States and Europe. Cryptococcal pericarditis in SOT recipients has, to our knowledge, only been reported in two lung transplantation recipients and a liver transplant recipient.^11–13^ We present the first case of isolated pericardial cryptococcal infection in a patient after heart transplant.

In SOT recipients, cryptococcosis is typically secondary to reactivation of latent infection from a subclinical pulmonary focus, usually occurring 16-21 months after transplantation.^14–18^ Early infection within the first few months after transplantation often occurs in lung or liver transplants due to undetected pre-transplant or donor-derived infection.^19^ De novo cryptococcal infection after transplant is also possible. Our patient became infected more than a year after transplant, thus we believe he developed cryptococcosis de novo or experienced reactivation of latent infection rather than donor-derived infection. High-dose steroids for thyroiditis possibly predisposed our patient to new infection or cryptococcus reactivation.

It is not clear why our patient had isolated pericardial involvement. Evaluation of his lungs and meninges revealed no evidence of cryptococcosis. There was no evidence of other infectious organ involvement or contiguous spread. Donor-derived infection is rare more than a year after transplantation, but possible. The donor, however, had no known high-risk exposures, was not immunosuppressed, and death was unrelated to infection. We suspect that our patient likely had a subclinical pulmonary focus, which in the context of increasing immunosuppressive medications, led to hematogenous seeding of the infection into his heart.

Optimal treatment for cryptococcosis in transplant recipients is based on expert opinion. There are currently no studies exploring cryptococcal infection in transplant recipients or in pericardial disease. Experts recommend extrapolating data from *Cryptococcus* infection from HIV patients.^1,20,21^ We treated the patient with 2-week induction therapy with L-Amb + flucytosine per Infectious Diseases Society and American Society of Transplantation guidelines.^1,21^ Our patient received 10 total doses of amphotericin over 3 weeks with flucytosine and unfortunately developed amphotericin-induced nephrotoxicity. He was transitioned to fluconazole to complete 12 months of maintenance therapy.

When feasible, immunosuppression should be lowered in patients with evidence of cryptococcosis.^1,21^ However, this needs to be balanced with the risk of organ rejection and immune reconstitution syndrome (IRIS). Calcineurin inhibitors are associated with decreased central nervous system involvement^17,22^ and mortality,^18^ attributed to their antifungal activity.^16^ However, discontinuation of calcineurin inhibitors has been linked with IRIS.^23^ Immunosuppressive agents should be gradually reduced, as more rapid reversal increases the risk of IRIS as well as organ rejection.^24,25^ In our patient’s case, tacrolimus and prednisone doses were slowly decreased, and serial endomyocardial biopsies were performed to assess for rejection. While there are no guidelines about how to monitor for recurrent infection, we obtained serial echocardiograms and serum cryptococcal antigens every 3 months.

This is one of the few reported cases of isolated *C neoformans* pericarditis following cardiac transplantation. Atypical manifestations of fungal infections, including cryptococcal pericarditis, should always be considered in SOT recipients presenting with unexplained findings. This case also highlights the complexities of cryptococcosis management among transplant recipients, notably the need to monitor for drug-induced toxicities and manage the delicate balance of decreasing immunosuppression, preventing organ rejection, while also avoiding IRIS.

## Figures and Tables

**TABLE 1:** Transplant cases

Transplant type	Brief description	Time since transplantation	Immunosuppression	Cryptococcal treatment	Procedures	Immunosuppressive tapering	Outcome	Reference
Liver	56-year-old M history of liver transplant. No other details of case reported	Unknown	Not reported	Ambisone and fluconazole (doses and duration not reported)	Pericardiocentesis, Pericardial window	Not reported	Clinical resolution	Dwivedi et al^13^
Lung	20-year-old F history of bilateral lung transplant for cystic fibrosis	8 mo	Not reported	Fluconazole 400 mg daily × 2 wk, then lost to follow up	Pericardial drainage	Not reported	Received 2 wk of therapy followed by clinical improvement and resolution	Levy et al^11^
Lung	68-year-old M history of mycobacterial avium complex and bilateral lung transplantation	9 mo	Tacrolimus 3 mg twice daily mycophenolate 250 mg daily prednisone 10 mg daily	L-Amb 3 mg/d, fluconazole 400 mg daily	Pericardiectomy planned	None. Same as on admission	Pericardiectomy is planned	El Helou and Hellinger^12^
Cardiac	52-year-old M history of orthotopic heart transplant due to genetic cardiomyopathy. Post-transplant rejection within 1 mo given antithymocyte. Thyroiditis treated with steroids 2 wk prior	15 months	Prednisone, tacrolimus, mycophenolate	L-Amp B 3 mg/kg + flucytosine × 3 wk, fluconazole × 12 months	Pericardiocentesis, Pericardial window and drain	Decreased prednisone, tacrolimus, and mycophenolate	Clinical improvement. Remains on fluconazole	This article

Abbreviations: F, female; L-Amp B, liposomal amphotericin B; M, male.

**TABLE 2:** Non-transplant cases

Immunosuppressed state, co-morbidities	Brief description	Cryptococcal treatment	Reference
AIDS	27-year-old M with AIDS and prior disseminated cryptococcal disease 3 mo earlier but no longer on medications	Lipo-Amp B + flucytosine	Brivet et al^5^
Hodgkin’s lymphoma	21-year-old M with hodgkin lymphoma and recent chemotherapy	Amb-B + Pericardiocentesis	Duvall et al^4^
Chronic myelomonocytotic leukemia, refractory to chemotherapy	71-year-old M with chronic myelomonocytic leukemia not improving with chemotherapy. Cultures became positive post-mortem	Not treated. On vancomycin, meropenem, micafungin and fluconazole.	Liu et al^7^
Intravenous drug use, possible AIDS	40-year-old F on therapy for presumed disseminated TB, possible AIDS (not confirmed), pigeon fancier	Amb-B + flucytosine and then weekly Amb-B + Pericardial window	Schuster et al^8^
Cryptococcal meningitis	25-year-old M on therapy for cryptococcal meningitis	Amphotericin, flucytosine, and itraconazole	Hsiao et al^9^
Metastatic prostrate cancer on chemo	66-year-old M with metastatic prostate cancer treated with leuprolide, docetaxel, prednisone, and carboplatin	Ampho-B + flucytosine	Ramey et al^6^
Feather refiner	27-year-old F who works as a feather refiner	IV fluconazole 400 mg daily × 4 wk, then oral fluconazole	Liu et al^10^

Abbreviations: Amb-B, amphotericin B; F, female; L-Amp B, liposomal amphotericin B; M, male.
